# Insecticide-impregnated netting: A surface treatment for killing *Lutzomyia longipalpis* (Diptera: Psychodidae), the vector of *Leishmania infantum*

**DOI:** 10.1016/j.crpvbd.2021.100044

**Published:** 2021-07-24

**Authors:** Vanessa de Araújo Barbosa, Cristian F. de Souza, Alisson Pereira, Derek Gatherer, Reginaldo P. Brazil, Daniel P. Bray, James G.C. Hamilton

**Affiliations:** a Laboratório Doenças Parasitárias, Instituto Oswaldo Cruz - Fiocruz, Pavilhão Arthur Neiva, Av. Brasil, 21040-360, Rio de Janeiro, RJ, Brazil; b Division of Biomedical and Life Sciences, Faculty of Health and Medicine, Lancaster University, Lancaster, LA1 4YG, UK; c Natural Resources Institute, University of Greenwich, Medway Campus, Central Avenue, Chatham Maritime, Kent, ME4 4TB, UK

**Keywords:** *Lutzomyia longipalpis*, *Leishmania infantum*, Sex-aggregation pheromone, λ-cyhalothrin, α-cypermethrin, (±)-9-methylgermacrene-B, Olyset plus, Interceptor

## Abstract

The sand fly *Lutzomyia longipalpis* is the main vector of *Leishmania infantum* in Brazil. Synthetic male-produced sex/aggregation pheromone co-located with micro-encapsulated λ-cyhalothrin in chicken sheds can significantly reduce canine infection and sand fly densities in a lure-and-kill strategy. In this study, we determined if insecticide-impregnated netting (IN) could replace insecticide residual spraying (IRS). We compared numbers of *Lu. longipalpis* attracted and killed in experimental and real chicken sheds baited with pheromone and treated with a 1 m^2^ area of either insecticide spray or netting. First, we compared both treatments in experimental sheds to control mortality established from light trap captures. We then compared the long-term killing effect of insecticide spray and netting, without renewal, in experimental sheds over a period of 16 weeks. Finally, a longitudinal intervention study in real chicken sheds compared the numbers and proportions of *Lu. longipalpis* collected and killed before and after application of both treatments. In Experiment 1, a higher proportion of males and females captured in IRS- and IN-treated sheds were dead at 24 h compared to controls (*P* < 0.05). No difference was found in the proportion of females killed in sheds treated with IN or IRS (*P* = 0.15). A slightly higher proportion of males were killed by IRS (100%) compared to IN (98.6%; *P* < 0.05). In Experiment 2, IN- and IRS-treated traps were equally effective at killing females (*P* = 0.21) and males (*P* = 0.08). However, IRS killed a significantly higher proportion of females and males after 8 (*P* < 0.05) and 16 (*P* < 0.05) weeks. In Experiment 3, there was no significant difference between treatments in the proportion of females killed before (*P* = 0.88) or after (*P* = 0.29) or males killed before (*P* = 0.76) or after (*P* = 0.73) intervention. Overall, initially the IN was as effective as IRS at killing female and male *Lu. longipalpis* in both experimental and real chicken sheds. However, the relative lethal effect of the IN deteriorated over time when stored under prevailing environmental conditions.

## Highlights

• Chicken sheds treated with netting or spray insecticide killed *Lutzomyia longipalpis.*
• Same effect was seen in experimental and real chicken sheds.
• Netting was as effective as spraying insecticide initially.
• Sprayed insecticide killed a higher proportion of both sexes after 8 and 16 weeks.
• The relative lethal effect of the netting deteriorated over time.

## Graphical abstract

**Figure:**
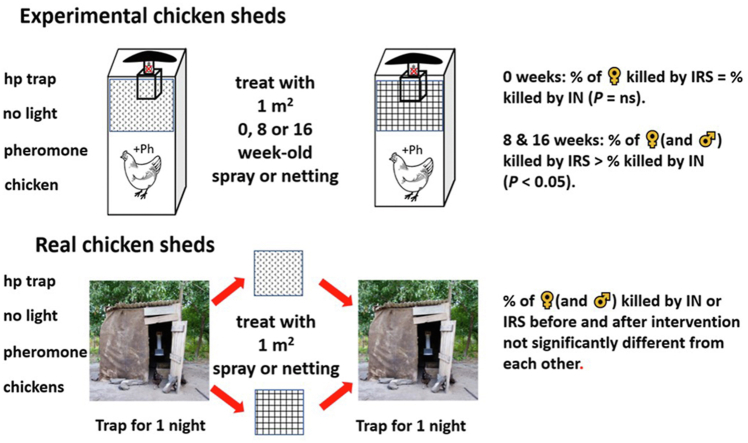

## 1 Introduction

Visceral leishmaniasis (VL) is an important neglected tropical disease around the world, with over 350 million people at risk of infection and an estimated 50,000 deaths per year (WHO, 2019). Brazil is one of six countries that have 90% of all reported VL cases. Between 2015 and 2017 more than 95% of the 11,000 reported human cases in South and Central America occurred in Brazil (Alvar et al., 2012; PAHO/WHO, 2018) and between 2007 and 2012 1,591 deaths caused by VL were recorded in Brazil (Donato et al., 2020). The disease, which is caused by infection with the protist parasite *Leishmania* (*Leishmania*) *infantum* (Kinetoplastida: Trypanosomatidae), is transmitted by the sand fly *Lutzomyia longipalpis* (Diptera: Psychodidae) from infected domestic dogs, *Canis familiaris* (Carnivora: Canidae) the reservoir host, to humans (PAHO/WHO, 2018; WHO, 2019).

Despite the vector control strategies adopted by the Brazilian Ministry of Health (MoH) over the past 20 years, the geographical range of *Lu. longipalpis* is spreading (Brazil, 2013; Casanova et al., 2015). The MoH sand fly control programme is reactive on human case detection: the home of the infected person, and any other human or animal dwelling within a 200 m radius, is sprayed with a residual insecticide (Ministério da Saúde, 2014). In addition, the MoH proactively monitors canine infections and when an infected dog is identified, it is euthanised (Dantas-Torres et al., 2012). These vector and infection control strategies have not reduced the incidence of the disease in dogs or humans (Courtenay et al., 2002; Costa, 2011; Harhay et al., 2011; Podaliri Vulpiani et al., 2011; Costa et al., 2013) and a recent analysis has shown that the burden of disease caused by VL more than doubled between 1990 and 2016 (Martins-Melo et al., 2018).

Spraying insecticide for sand fly control is challenging for local health authorities because of the cost and effectiveness of the activity. The Brazilian MoH recommend that insecticide spraying must be repeated three to four months after the initial treatment (Ministério da Saúde, 2014). In addition, to ensure effectiveness of insecticide application and to avoid the development of resistance in the vector, residual insecticide spraying requires trained operatives with appropriate infrastructure to ensure well-maintained spraying equipment and an effective application regime (Alexander et al., 2009; Chowdhury et al., 2011).

Male and female *Lu. longipalpis* form aggregations on or near host animals for mating and female blood-feeding with chicken sheds being a common aggregation site in peridomestic environments (Kelly and Dye, 1997). Although it is not clear why some aggregation sites are favoured over others, aggregation behaviour is largely driven by the male produced sex-aggregation pheromone (Spiegel et al., 2016; Bell et al., 2018; Retkute et al., 2021). The use of insecticide has a disruptive effect on *Lu. longipalpis* aggregation formation. Those males that arrive first at an insecticide-treated site are killed, and any further pheromone mediated recruitment of females and males is stopped (Kelly et al., 1997). A consequence of this disruption is that new sand fly aggregations are more likely to occur at sites that have not been treated with insecticide (Kelly et al., 1997; Bray et al., 2010). In practice, this means that most of the insecticide used as a long-term *Lu. longipalpis* vector control tool is wasted (Kelly et al., 1997). The use of synthetic sex-aggregation pheromone in insecticide-treated sites overcomes the disruptive effect of the insecticide by continuing to attract female and male sand flies (Bray et al., 2009, 2010) and a controlled release formulation of the pheromone can attract *Lu. longipalpis* for up to 12 weeks (Bray et al., 2014) greatly extending the lethal effect of the insecticide (Gonzalez et al., 2017, 2019). A trial of the synthetic sex-aggregation pheromone, (*±*)-9-methylgermacrene-B (Krishnakumari et al., 2004), formulated in a long-lasting controlled release device (Bray et al., 2009, 2014) co-located with sprayed microencapsulated λ-cyhalothrin (Demand CSW; BASF PLC, Cheshire, UK) in chicken roosting sites significantly reduced *Lu. longipalpis* densities, canine *Leishmania* parasite infection incidence, tissue loads and canine seroconversion incidence and established the potential for this strategy to reduce disease incidence (Courtenay et al., 2019; Gonçalves et al., 2021; Retkute et al., 2021).

Results of a previous laboratory study indicated that Blue Olyset netting (Sumitomo Chemical Co. Ltd., Tokyo, Japan), impregnated with 2% permethrin, could be an effective replacement for sprayed microencapsulated λ-cyhalothrin (20 mg a.i./m^2^) (WHO, 2017). As insecticide-impregnated netting can remain active for several years and is widely available as the main intervention against malaria transmission, its use in *Lu. longipalpis* control could overcome some of the current challenges of residual insecticide spraying (staff training, dose control, cost, efficacy, incidental environmental contamination and support infrastructure) (Bray and Hamilton, 2013). In addition, residents can sometimes refuse the application of the insecticide spray because of the damage that it causes to the walls of their homes.

The aim of the present study was to determine if insecticide-impregnated netting could be an effective replacement for IRS for killing female and male *Lu. longipalpis* under field conditions. Thus, our objectives were to compare the killing effect of the two treatments when first applied and up to 16 weeks later in experimental sheds, and then to compare the relative efficacy of the two treatments when applied in real chicken sheds.

We compared the lethal effect of α-cypermethrin-impregnated netting (Interceptor, BASF Chemical Co.) with λ-cyhalothrin residual spray in experimental chicken sheds and permethrin (2%) + piperonyl butoxide (1%)-impregnated netting (Olyset Plus, Sumitomo Chemical UK PLC) with α-cypermethrin residual insecticide spray in real chicken sheds. It was not our intention to compare the efficacy of the insecticides, instead our study was to compare whether the mode of delivery of the insecticides (spray or impregnated netting) had an effect on mortality.

## 2 Materials and methods

### 2.1 Study site

The study took place in Governador Valadares (GV), a municipality of approximately 280,000 people in Minas Gerais State, Brazil (18°51′W, 41°57′S, altitude 170 m) 320 km northeast of Belo Horizonte, the state capital. This area is a focus of intense VL transmission and is also endemic for cutaneous leishmaniasis where the sand fly vector, *Lu. longipalpis*, is abundant (Barata et al., 2013; Valdivia et al., 2017). GV is situated in the Rio Doce basin an area where the local topography consists of valleys and hills, and which was originally covered by dense ombrophilous forests (Atlantic Forest) but which is now heavily modified by anthropic intervention (Fernandes Filho and Schaefer, 2002). The climate is the Aw type (tropical sub-warm and sub-dry) according to the Köppen-Geiger classification (Peel et al., 2007). GV has an average temperature of 24.2 °C, (range 15.2–33 °C) and an average annual rainfall of 1,109 mm concentrated between October and March (Climate-data.org, 2017).

Experiments were carried out in the private gardens and yards of volunteer householders. The dates of trapping and location of the sites are summarised in the Supplementary Table S1. Typically, the householder's gardens consisted of a walled-in area at the front or back of the property which contained fruit trees, shrubs, mature trees and animal shelters. Experiments 1 and 2 were carried out in experimental chicken sheds (Bray et al., 2010) in the Vila Parque Ibituruna neighbourhood, and Experiment 3 was carried out in householders' own chicken sheds in the Vila Isa and Vilage da Serra, neighbourhoods. These neighbourhoods are typical Brazilian peri-urban areas, with homes built near the Área de Preservação Ambiental (APA) Ibituruna forest reserve.

The inclusion criteria for all experiments were that the households had a yard containing a chicken shed with chickens and that *Lu. longipalpis* were present. Presence of *Lu. longipalpis* was confirmed through preliminary sampling in the householder's chicken shed. Solvent (hexane) extracts of individual sand flies collected in GV both prior to and during field experiments were examined by coupled gas chromatography-mass spectrometry (GC/MS) (Hamilton et al., 2005) to confirm that they produced (*S*)-9-methylgermacrene-B sex-aggregation pheromone.

Taxonomic identification of sand fly species and their sex (male or female) for all experiments was by microscope (Nikon SMZ 445) examination of morphological characteristics. Male *Lu. longipalpis* were initially identified by the presence of a pale spot on abdominal tergite IV and then confirmed by the morphological characteristics of the genitalia (Mangabeira Filho, 1969; Galati, 2003). Females were dissected and the cibarium and spermathecae examined to confirm species identification (Galati, 2003).

### 2.2 Chicken sheds

**Fig. 1:**
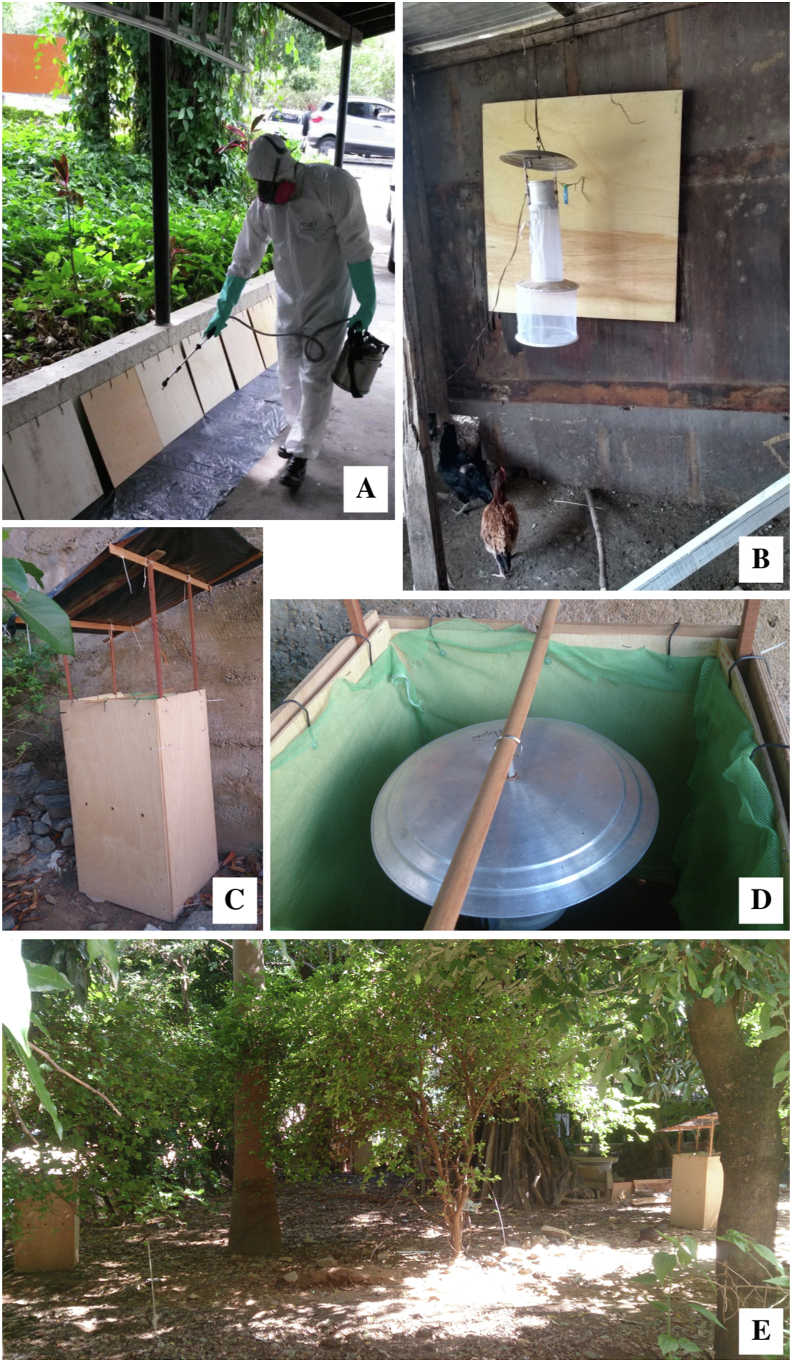
Photographic images illustrating the chicken sheds and insecticide treatments used in experiments 1, 2 and 3. **A** Spray application of insecticide on wooden panels used in experimental chicken sheds in Experiments 1 and 2. **B** Insecticide-sprayed wooden panel placed in the real chicken shed in Experiment 3. **C** Specially constructed experimental chicken shed used in Experiments 1 and 2. **D** Insecticide-impregnated netting covered wooden panels *in situ* within in the experimental chicken shed used in Experiments 1 and 2. **E** Position of a pair of experimental chicken sheds located in household A used in Experiments 1 and 2.

To allow direct comparison of numbers of sand flies in the different treatment in Experiments 1 and 2 we standardised the design of the chicken sheds by using specially constructed experimental chicken sheds. These were constructed from 4 plywood panels each measuring 105 cm high × 55 cm wide arranged in a square plan (55 × 55 cm) (Fig. 1). The panels were held together by plastic cable-ties passed through holes (10 mm diameter) in the top and bottom corners of each panel (Bray et al., 2010). Sand flies were collected in miniature suction traps manufactured in Brazil (Hoover Pugedo (HP)) (Pugedo et al., 2005). The light bulb was removed from the trap and instead a pheromone lure, containing 10 mg of synthetic sex pheromone ((±)-9-methylgermacrene-B), was attached to the underside of the lid of each trap (Bray et al., 2014). The trap was suspended inside the experimental chicken shed from a wooden dowel (20 mm in diameter) placed across the top of the shed. Sand flies were collected in a nylon Barraud cage (22 × 22 × 22 cm) suspended below the HP trap. A chicken, supplied with food and water, from the household flock was placed on the ground inside the experimental chicken shed overnight.

The chicken sheds used in Experiment 3 belonged to the householders and were constructed primarily out of locally available recycled wood but also included corrugated metal, asbestos sheet and plastic. The primary function of these real chicken sheds was to shelter the chickens from nocturnal predators and thus they had walls, a roof and a door but insects could enter and leave freely. Once closed after dusk the chickens remained in the shed throughout the night until they were released by the householder in the morning. The sheds used in the study were selected based on their size (range 1–10 m^2^) and the number of chickens that they contained (5–30).

### 2.3 Insecticide treatment

For Experiments 1 and 2, experimental chicken sheds were treated with either insecticide-impregnated netting or an insecticide spray. For the netting treatment, a single layer of netting (Interceptor®, BASF S.A., São Paulo, Brazil) which is impregnated with α-cypermethrin (6.7 g/kg or 200 mg/m^2^) during manufacture was fixed onto plywood panels (0.5 × 0.5 m) and four of these were fitted on the inside at the top of the 4 interior walls of the experimental chicken shed (Fig. 1). The total area covered was 1 m^2^. For the insecticide spray treatment, 4 plywood panels (0.5 × 0.5 m) were sprayed with microencapsulated λ-cyhalothrin (Karate Zeon 50 CS, Syngenta, Huddersfield, UK; 20 mg a.i./m^2^) which were then fitted inside at the tops of the 4 walls of the experimental chicken shed. The total area treated was 1 m^2^ and was applied at the dosage required by the Brazilian MoH VL control handbook (Ministério da Saúde, 2014).

For Experiment 3, a single layer of Olyset® Plus polyethylene netting (Sumitomo Chemical Company UK PLC, London, UK) impregnated with permethrin [2.0% w/w (20 ± 5 g/kg)] and piperonyl butoxide (PBO) [1.0% w/w (10 ± 2.5 g/kg)] during manufacture was fixed onto a plywood panel (1 m × 1 m) and placed inside the real chicken shed or a plywood panel (1 m × 1 m) sprayed with α-cypermethrin (Alfatek 200 SC, Rogama) (20 mg/m^2^) (Ministério da Saúde, 2014) was placed inside the chicken shed (Fig. 1).

The netting and spray treatments used in the experimental chicken sheds were different to those used in the real chicken shed experiments because we were only able to use those insecticide treatments (spray and net) that were available to the project at the time.

There was no record of recent insecticide use at any of the study sites.

### 2.4 Experimental design

#### 2.4.1 Experiment 1

To assess the lethal effect of insecticide-treated netting and insecticide spray on female and male *Lu. longipalpis*, we compared the numbers of sand flies caught, and the proportion which were dead at 24 h, in HP suction traps suspended in netting- or spray-treated experimental sheds and in control traps. Collections were made in the experimental sheds and control traps over 4 nights at 2 houses, A and B. Two pairs of experimental sheds were used at each house and the position of the netting- and spray-treated sheds within each pair was swapped between replicates to control for positional bias in sand fly numbers. The trapping cycle was repeated on subsequent nights. The experimental design gave a total of 32 possible trap catch data points from 8 replicates. However, replicates were excluded if a complete set of data was not obtained for the replicate (for example if one or more of the traps did not function correctly on any pair of nights) thus we obtained 28 trap-catch data points from 7 experimental replicates.

Experimental chicken sheds were placed in the yards of each of the two houses (A and B) prior to sunset (approximately 18:00 h) (Fig. 1). The experimental chicken sheds in each pair were 3 m apart and the 2 pairs in each household were 5 m from each other. The two households were 25 m apart. Each pair of experimental chicken sheds consisted of one fitted with sprayed insecticide-treated wooden panels and the other one fitted with insecticide-impregnated netting-treated wooden panels.

The following morning (approximately 14 h after the traps were placed) the HP traps and attached Barraud cages were removed and the chickens released. The number of live and dead female and male *Lu. longipalpis* sand flies in each Barraud cage were then counted. The numbers of blood-fed females caught were very low and therefore not included in the analysis.

The live sand flies were transferred to plastic holding pots (9.5 cm in diameter, 8 cm deep) with a nylon netting top. The base of the holding pot had previously been filled with Plaster of Paris (0.5 cm deep) and was dampened to maintain humidity. A piece of cotton wool, soaked in a solution of 20% sucrose and 50% honey syrup, was placed on the top of each holding pot as a sugar source for the sand flies. The pots were then placed on a layer of moistened filter paper in the bottom of a Styrofoam box (28 cm L × 24 cm W × 35 cm D), covered with a dark cloth and kept for an additional 10 h after which time the number of live and dead, male and female sand flies in each pot were counted again.

Control HP traps with a tungsten light only, were placed beside the real chicken sheds of each of the 2 study houses used in Experiment 1 and 2. The collected alive and dead sand flies were counted, and the live insects placed in holding pots for an additional 10 h after which their mortality was recorded in the same way as for those exposed to the different insecticide treatments. To avoid causing mortality through handling, species identity was determined at the end of the experiment and only *Lu. longipalpis* numbers were tabulated. Thus, we recorded the total number of male and female sand flies collected as well as the total number of males and females that were dead 24 h after the traps had been placed for both insecticide treatments and controls.

#### 2.4.2 Experiment 2

To compare the lethal effect of insecticide-treated netting with residual spraying over time (16-weeks), two pairs of experimental chicken sheds were placed at each of the 2 houses used in Experiment 1. Trapping was performed over 4 nights at each of 3 timepoints; week 1 (May), week 8 (July) and week 16 (September). In total 8 replicates were performed at each time point. However, as before, replicates were excluded from the analysis if they were partially completed (due to trap failure, for example). Thus, we had 6 replicates in May, 7 replicates in July and 8 replicates in September (i.e. 21 replicates (84 data points) in total).

When not in use, the insecticide-treated panels were removed from the experimental chicken sheds and kept uncovered and thus exposed to the prevailing weather conditions.

#### 2.4.3 Experiment 3

To compare the effect of sprayed insecticide with netting insecticide treatments on *Lu. longipalpis* in real chicken sheds, a longitudinal intervention study (Bray et al., 2010) was carried out in a 9-week period during August and September 2019. We used 4 chicken sheds; A, B, C and D. Sheds A and B each had 3 replicates of the spray treatment and 2 replicates of the netting treatment while sheds C and D each had 3 replicates of the netting treatment and 2 replicates of the spray treatment. The distances between experimental sites varied from 82 to 3,110 m.

On the first night of the experiment an HP trap (without a light) and a pheromone lure (*c.*30 cm from the trap) was placed inside each of the chicken sheds at 6 pm. The trap and pheromone remained in position in the chicken shed overnight. The next morning, approximately 12 h later, the cages containing the sand flies collected overnight were removed, and the number of sand flies (male and female, dead and alive) was recorded. Live sand flies were removed from the collection cage and placed in a pot and held for a further 12 h. On the evening after the first night of trapping a 1 m^2^ (1 × 1 m) insecticide-treated wooden board (treated with either Olyset Plus netting or α-cypermethrin spray) was added to the chicken shed alongside the HP trap and pheromone (Fig. 1). A fresh collection cage was attached to the HP trap. The next morning the overnight sand fly collection was removed, and sand flies processed as before. The insecticide treatment, pheromone lure and HP trap were also removed from the chicken shed. The insecticide treatments were freshly prepared for each week of trapping.

Thus, at the end of the period, we recorded the total number of male and female sand flies collected as well as the total number of males and females that were dead after 24 h both before and after the application of the intervention.

Chickens were present throughout each experiment (mean ± standard error (SE); 20.0 ± 3.7 per shed). Five experimental replicates were carried out at each chicken shed with an interval of at least 7 days between replicates.

### 2.5 Data analysis

Histograms of the data did not follow a parametric distribution and therefore non-parametric tests were applied.

#### 2.5.1 Experiment 1

The aims of statistical analysis for Experiment 1 were to determine if there were significant differences in the numbers of *Lu. longipalpis* caught and killed in control traps and traps in experimental chicken sheds treated with insecticide netting and spray. Male and female *Lu. longipalpis* were analysed separately. Kruskal-Wallis tests were used to compare numbers of sand flies caught between the three treatments. Where a significant overall effect was found, Wilcoxon pairwise tests with *post-hoc* correction (*P* < 0.05) were applied to identify significant differences between individual treatments. The same procedure was used to compare the proportions of *Lu. longipalpis* collected which were dead at 24 h between treatments. All statistical analyses were conducted in R v1.3 (Wickham, 2016; R Development Core Team, 2020).

#### 2.5.2 Experiment 2

The aims of statistical analysis for Experiment 2 were to determine if the numbers of sand flies caught, and the proportion killed after 24 h, in spray- and netting-treated sheds varied over time. Effects on male and female *Lu. longipalpis* were analysed separately. A Kruskal-Wallis test was used to compare numbers of sand flies caught by each treatment in each month (six treatment by month combinations). Where a significant overall effect was found, pairwise Wilcoxon tests with a correction for multiple comparisons were applied to test for differences in numbers of sand flies caught between months, and then between treatments in each month. The same procedure was used to compare the proportion of sand flies captured which were dead at 24 h between treatments and months.

#### 2.5.3 Experiment 3

Analyses of data collected in Experiment 3 aimed to determine whether there was a difference in the effects of insecticide netting and spray on the numbers of sand flies captured in real chicken sheds, and the proportion collected which were dead at 24 h. Mann-Whitney U-tests were used to determine if numbers of sand flies captured differed between treatments, both before and after application. For each replicate, change in the numbers of sand flies captured with each treatment application were then calculated as number captured after treatment minus numbers collected before treatment. Mann-Whitney U-tests were then used to determine if the change in numbers of sand flies caught differed between netting and spray insecticide treatments. One-sample Mann-Whitney U-test was used to test whether numbers of sand flies captured before treatment were significantly different to numbers caught after treatment (i.e. was the overall change significantly different from zero). Wilcoxon signed-rank tests were used to determine if the proportion of captured sand flies dead at 24 h was significantly different before treatment compared to after treatment. Mann-Whitney U-tests were used to compare the proportions killed between the netting and spray treatments, before and after application.

## 3 Results

### 3.1 Species identification

Both *Lu. longipalpis* (98.4%) and *Evandromyia cortelezzii* (1.6%) were trapped in the control traps placed in houses A and B; however, only the numbers of *Lu. longipalpis* are reported (Supplementary Table S2).

### 3.2 Experiment 1

**Fig. 2:**
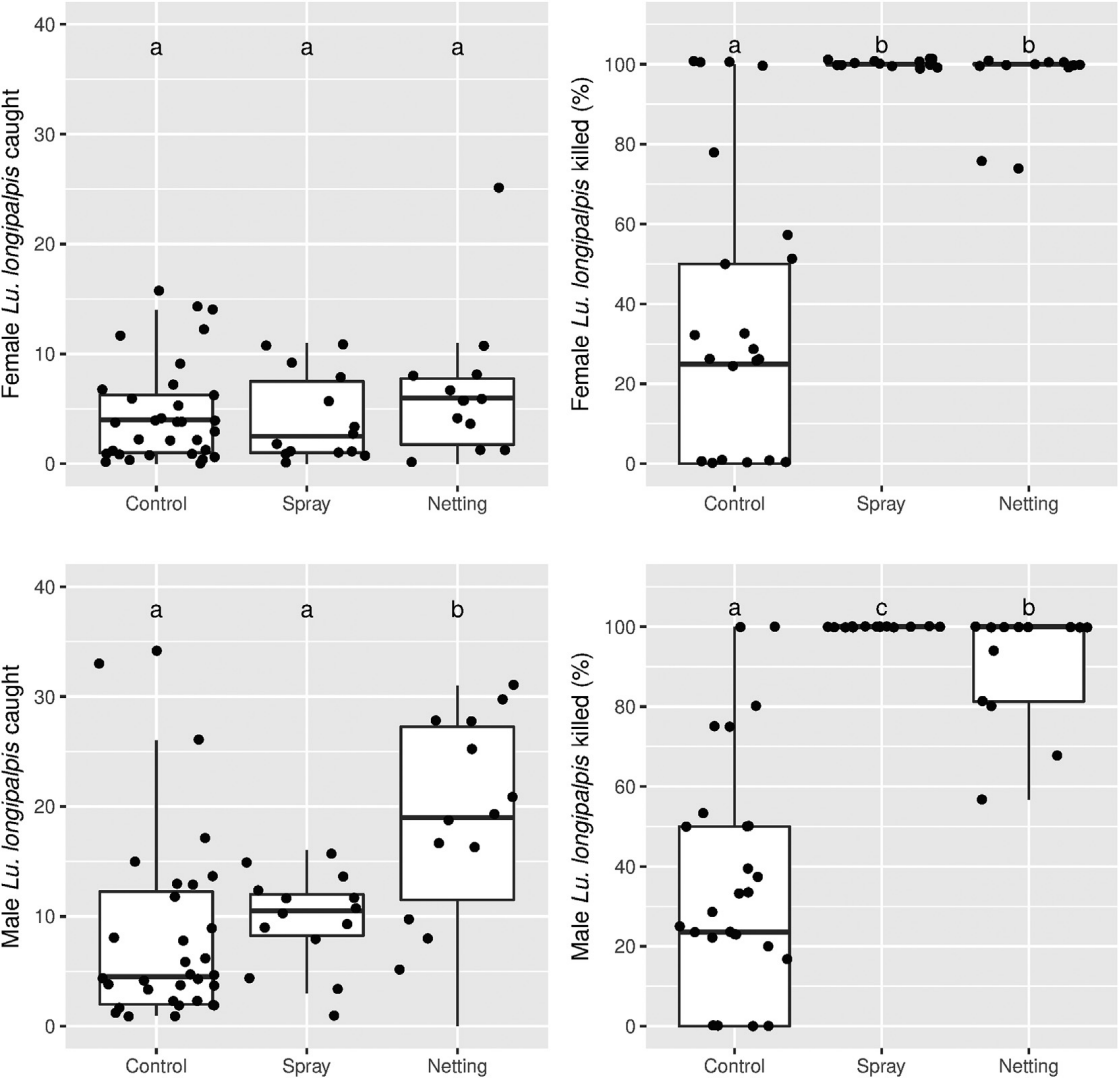
Number of female (top) and male (bottom) *Lutzomyia longipalpis* caught (left) and percentage dead (right) at 24 h in Hoover Pugedo suction traps. Data are superimposed over boxplots (median, 25–75% quantiles). Traps were placed next to real chicken sheds (“Control”) or in experimental chicken sheds treated with λ-cyhalothrin spray (“Spray”) or α-cypermethrin-impregnated netting (“Netting”). Different letters (a-c) indicate significant differences between treatments (pairwise Wilcoxon test, *P* < 0.05).

In total 538 (mean ± SE; 9.6 ± 1.1) *Lu. longipalpis* [393 (14.0 ± 1.6) males and 145 (5.2 ± 1.0) females] were collected in 12 nights of trapping effort in both spray- and netting-treated experimental chicken sheds (Fig. 2; Supplementary Table S3). The overall ratio of males:females was 2.7:1. Trapping data for night 1 (N1) house A1 and night 2 (N2) house A1 was not included because the CDC trap failed to operate on N1.

*Females*: Overall, there was no significant effect of treatment (netting, spray, control) on number of females caught (Kruskal-Wallis test, χ^2^ = 0.85, *df* = 2, *P* = 0.65; Fig. 2, top left). However, there was a significant difference between treatments in the proportion of females caught that were dead at 24 h (Kruskal-Wallis test, χ^2^ = 32.4, *df* = 2, *P* < 0.001; Fig. 2, top right).

A higher proportion of females were dead at 24 h in the spray-treated experimental chicken sheds (100%) compared to the control traps (31.4%) (Wilcoxon pairwise test adjusted for multiple comparisons, *P* < 0.001). Similarly, a higher proportion of females were dead at 24 h in the netting-treated experimental chicken sheds (93.4%) compared to controls (Wilcoxon pairwise test, *P* < 0.001). No difference was found in the proportion of females dead at 24 h in boxes treated with netting or spray (*P* = 0.15).

*Males*: Overall, there was a significant effect of treatment (netting, spray, control) on the number of males caught (Kruskal-Wallis test, χ^2^ = 11.9, *df* = 2, *P* < 0.01; Fig. 2, bottom left). More males were caught in experimental chicken sheds treated with netting than in the sprayed experimental chicken sheds (Wilcoxon test, *P* < 0.05) or the control traps (Wilcoxon test, *P* < 0.01). No difference was found between numbers caught in spray-treated experimental chicken sheds or control traps (Wilcoxon test, *P* = 0.13)

A significant difference was also observed between the three treatments in the proportion of males that were dead at 24 h (Kruskal-Wallis test, χ^2^ = 39.5, *df* = 2, *P* < 0.001; Fig. 2, bottom right). A higher proportion of males dead at 24 h was found in the spray treatment (100%) when compared to the netting treatment (98.6%; Wilcoxon test, *P* < 0.05) and control traps (23.6%; Wilcoxon test, *P* < 0.001). The proportion of males dead at 24 h in the netting-treated boxes was also higher than in the control traps (Wilcoxon test, *P* < 0.001).

The results of this experiment suggest that spraying and netting treatments were equally effective at killing females although spraying may be more efficient at killing males than netting. However, as a significantly greater number of males were caught in netting-treated sheds (257 *vs* 136, Wilcoxon signed-rank test, *P* < 0.05) the netting-treated sheds therefore produced the greatest male mortality (225 *vs* 136; Wilcoxon signed-rank test, *P* < 0.05).

### 3.3 Experiment 2

In total 2,034 (mean ± SE; 12.1 ± 1.0) *Lu. longipalpis* [1,441 (17.5 ± 1.8) males and 593 (7.1 ± 0.6) females] were collected during 42 nights trapping effort in three 1-week trapping periods in May, July and September (Supplementary Table S4). The overall ratio of males:females was 2.4:1 and significantly more males than females were caught in both the netting- and spray-treated experimental chicken sheds (Wilcoxon signed-rank test, *P* < 0.001 for both).

**Fig. 3:**
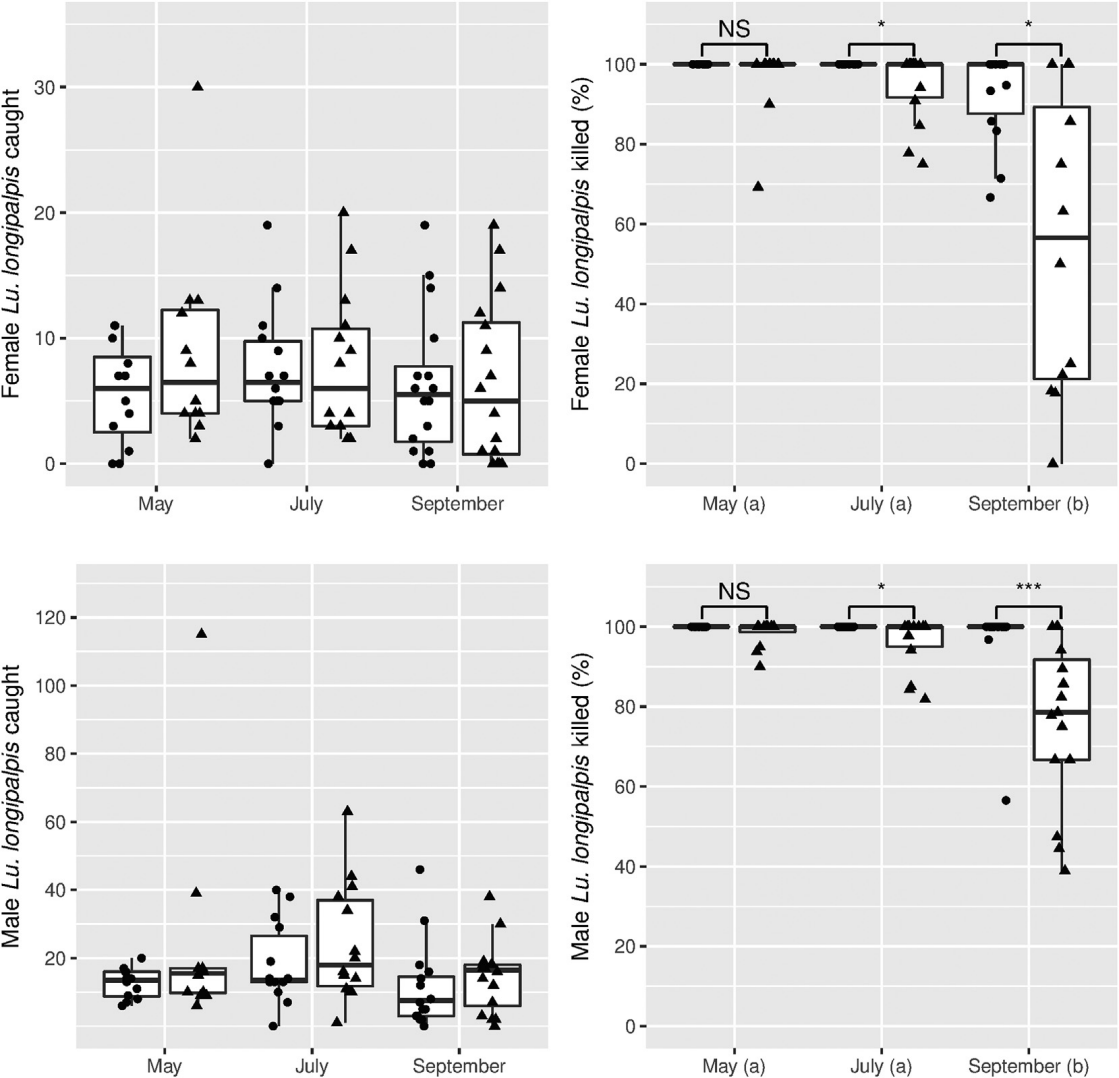
Number of female (top) and male (bottom) *Lutzomyia longipalpis* caught (left) and percentage dead (right) at 24 h in Hoover Pugedo suction traps. Data are superimposed over boxplots (median, 25–75% quantiles). Traps were in experimental chicken sheds treated with λ-cyhalothrin spray (circles) or α-cypermethrin-impregnated netting (triangles). Different letters (a, b) indicate significant differences between percentage of sand flies killed between months (pairwise Wilcoxon test, *P* < 0.05). Asterisks indicate significant differences between treatments within months (pairwise Wilcoxon test: ∗*P* < 0.05; ∗∗*P* < 0.01; ∗∗∗*P* < 0.001; NS, not significant). No overall effect of month and treatment was found on numbers of female and male *Lu. longipalpis* caught (Kruskal-Wallis test).

*Females*: There was no overall significant difference in numbers of females caught across treatments and time points (Kruskal-Wallis test, χ^2^ = 2.5, *df* = 5, *P* = 0.78; Fig. 3, top left). However, there was a significant overall difference across treatments and timepoints in the proportion of females dead at 24 h (Kruskal-Wallis test, χ^2^ = 28.0, *df* = 5, *P* < 0.001; Fig. 3, top right). When both treatments (netting and spray) were combined a significantly higher proportion of females were killed in May and July than in September (Wilcoxon pairwise test, *P* < 0.01). No significant difference was found between proportions of females killed in May and July (*P* = 0.4). In comparisons between treatments in each month, no significant difference was found in the proportions of females killed by spray or netting in May (Net:Spray 0.93 *vs* 1) (Wilcoxon pairwise test, *P* = 0.21). However, spray killed a significantly higher proportion of females than netting in July (0.93 *vs* 0.99) (*P* < 0.05) and September (0.50 *vs* 0.91) (*P* < 0.05).

*Males*: There was no overall significant difference in numbers of males caught across treatments and time points (Kruskal-Wallis test, χ^2^ = 8.6, *df* = 5, *P* = 0.13; Fig. 3, bottom left). However, there was a significant overall difference across treatments and timepoints in the proportion of males dead at 24 h (Kruskal-Wallis test, χ^2^ = 36.1, *df* = 5, *P* < 0.001; Fig. 3, bottom right). When both treatments (netting and spray) were combined, a significantly higher proportion of males were killed in May and July than in September (Wilcoxon pairwise test, *P* < 0.01). No significant difference was found in the proportion of males killed in May and July (*P* = 0.5). In comparisons between treatments in each month, no significant difference was found in the proportion of males killed by spray and netting in May (Net:Spray 0.99 *vs* 1) (Wilcoxon pairwise test, *P* = 0.08). However, spray killed a significantly higher proportion of males than the netting in July (0.95 *vs* 0.99) (*P* < 0.05) and September (0.70 *vs* 0.89) (*P* < 0.001).

The results suggest that initially (during May) the α-cypermethrin-impregnated netting and λ-cyhalothrin residual spray were equally good at killing both female and male sand flies. However, the effectiveness of the netting treatment deteriorated over time so that in July and September significantly fewer male and female sand flies were killed by the netting compared to the spray treatment.

### 3.4 Experiment 3

In total 1,073 (mean ± SE; 13.4 ± 1.7) *Lu. longipalpis* [788 (19.7 ± 2.9) males and 285 (7.1 ± 0.9) females] were collected before and after intervention during a 20-night period from August to September (Supplementary Table S5). The overall ratio of males:females was 2.8:1.

In the chicken sheds treated with α-cypermethrin-impregnated netting, 519 (mean ± SE; 13.0 ± 2.7) *Lu. longipalpis* in total [395 males (19.8 ± 4.9) and 124 (6.2 ± 1.1) females] were collected before and after the intervention. In the chicken sheds treated with λ-cyhalothrin spray treatment, 554 (mean ± SE; 13.9 ± 2.0) *Lu. longipalpis* in total [393 (19.7 ± 3.3) males and 161 (8.1 ± 1.4) females] were collected before and after the intervention. The numbers of *Lu. longipalpis* (males and females) were similar for both the netting- and spray-treated elements of the experiment.

**Fig. 4:**
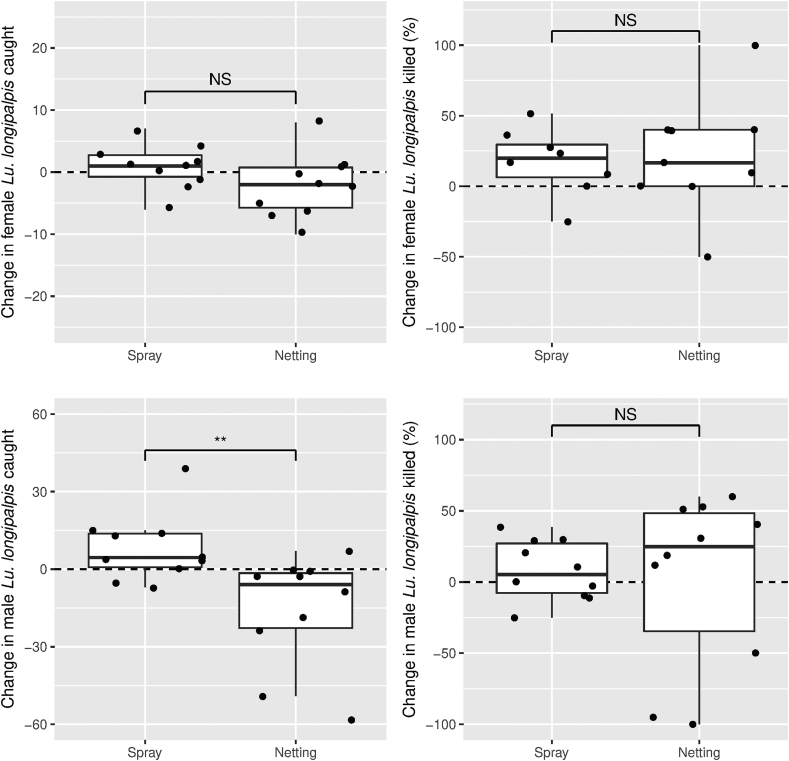
Change in number of female (top) and male (bottom) *Lutzomyia longipalpis* caught (left) and percentage dead (right) at 24 h in Hoover Pugedo suction traps. Data are superimposed over boxplots (median, 25–75% quantiles). Traps were in real chicken sheds treated with permethrin spray (“Spray”) or α-cypermethrin-impregnated netting (“Netting”). Asterisks indicate significant differences between treatments (Mann-Whitney U-test: ∗*P* < 0.05; ∗∗*P* < 0.01; ∗∗∗*P* < 0.001; NS, not significant). There was a significant overall effect of treatment on percentage of females killed (top right, Wilcoxon test, *P* < 0.05) but not males killed (bottom right, Wilcoxon test, *P* > 0.05).

*Females*: Overall, no significant difference was found between sheds treated with netting or spray in the change in number of females captured before and after treatment (Mann-Whitney U-test, *W* = 28, *P* = 0.10; Fig. 4, top left). The numbers of females captured before treatment was not significantly different to the numbers captured after the two treatments were applied (One sample Mann-Whitney U-test, *V* = 73, *P* = 0.60).

However, the treatments had a significant effect on the change in proportion of females that were dead at 24 h after treatment compared to before treatment (Wilcoxon pairwise test, *V* = 18, *P* < 0.05; Fig. 4, top right). Prior to treatment the median proportion of female sand flies dead at 24 h was 0.6 (interquartile range = 0.33–0.83) but after the treatment the proportion dead at 24 h was 0.73 (0.61–1.0). There was no significant difference between the treatments in the proportion of females dead at 24 h before treatment (Mann-Whitney U-test, *W* = 38.0, *P* = 0.88) or after treatment (Mann-Whitney U-test, *W* = 63.5, *P* = 0.29).

Therefore, both treatments increased female sand fly mortality after application and were not found to be significantly different from each other in their effectiveness.

*Males*: A significant difference was found between sheds treated with netting or spray in the change in numbers of males caught following application (Mann-Whitney U-test, *W* = 14.5, *P* < 0.01). The numbers of males captured dropped significantly following application of netting (one-sample Mann-Whitney U-test, *V* = 4, *P* < 0.05), but not of spray (*V* = 36.5, *P* = 0.1) (Fig. 4, bottom left).

There was no difference between treatments in the proportion of males dead at 24 h prior to application (Mann-Whitney U-test, *W* = 54.5, *P* = 0.76) or after application of the interventions (*W* = 55, *P* = 0.73) (Fig. 4, bottom right). Considering both treatments together, no difference was found in the proportion of males collected that died at 24 h before and after treatment: the treatments had no significant effect on male sand fly mortality (Wilcoxon test, *V* = 66, *P* = 0.25).

In summary, both the netting and spray treatments increased the proportion of females killed but had no effect on the proportion of males killed. No difference was found between the effectiveness of netting and spray treatments in killing female sandflies. The netting treatment reduced the numbers of males captured, but not the number of females. The spray and netting treatments had no effect on the number of females caught.

## 4 Discussion

Insecticide-impregnated netting is widely available for use for malaria vector control and although there are concerns over its misuse (Berthe et al., 2019) the potential of insecticide-impregnated materials including netting for insect control has been recognised and evaluated for use in crop protection (Dáder et al., 2012; Marianelli et al., 2019). The potential repurposing of insecticide-impregnated netting as a surface treatment to replace sprayed insecticide treatments for vector control was previously investigated in a laboratory study (Bray and Hamilton, 2013) and the present study is a further evaluation of the potential of netting as a surface treatment in a field setting.

The results of Experiment 1 indicated that the IRS and IN treatments were equally effective at killing females in experimental chicken sheds, but IRS was slightly more effective at killing males. The proportion of females dead at 24 h was not significantly different in IRS-treated sheds compared to the IN-treated sheds. The mortality in both spray- and netting-treated sheds was higher than the control traps.

By contrast, significantly more males were caught and dead at 24 h in the IN-treated sheds compared to the IRS-treated sheds and a higher proportion of males in the IRS-treated sheds were dead at 24 h compared to the IN-treated sheds. The differential effect of insecticide on males compared to females has previously been observed in other studies (Kelly et al., 1997; Feliciangeli et al., 2003). The difference has been associated with the relatively small size of the males making them more susceptible to the insecticides than females (Feliciangeli et al., 2003). However, it may also be that in our experiments these differences are related to the different behaviours of male and female *Lu. longipalpis* in the proximity of hosts and thus their exposure time to the insecticide treatments. Males rest on the surfaces near the blood-meal source where they defend territories and when females enter these territories, they mate (Morrison et al., 1995; Kelly and Dye, 1997). Thus, the males are likely to be in contact with the insecticide-treated surfaces for longer than the females and thus may be disproportionately affected by the insecticide. Our study only accounted for those *Lu. longipalpis* that entered the suction traps and did not account for the sand flies that might have been affected by insecticide before entering the trap. Given the irregular construction of the real chicken sheds and the multiple opportunities for evasion of capture after exposure to insecticide, the methodology utilised in other studies such as placing sheeting on the floor (Del Rio et al., 2014) would seem impractical. However, counting the dead sand flies not found inside the HP traps could potentially provide very useful information in the experimental chicken shed trials if the collecting sheet could be separated in some way from the chicken.

Another possible explanation for the reduced numbers of *Lu. longipalpis* collected in traps placed in chicken sheds (both experimental and real) treated with spray rather than netting may be related to the relative repellent effect of the insecticides, i.e. the spray treatment moved sand flies away from the HP traps. Repellence can be an advantage of pyrethroids, which is useful in reducing insect contact with the individual and thus offering personal protection against bites (Kawada et al., 2014). However, in a community vector control programme, the repellent effect of spraying may be to divert the sand flies away from treated to untreated surfaces. The use of synthetic sex aggregation pheromone in these studies would however have overcome the repellent effect of the pyrethroid insecticides (Bray et al., 2010).

In Experiment 2 no significant differences were found in the proportion of females killed by netting or spray when first applied. However, spray killed a higher proportion of females than netting in July and September. The same pattern was also observed for males.

The deterioration of effectiveness of the α-cypermethrin netting relative to the λ-cyhalothrin spray treatment was likely related to changes in the effectiveness of the netting rather than a change in the response of the sand flies over time. However, it is unclear why the α-cypermethrin netting became less effective over time in these experiments. In a previous laboratory study λ-cyhalothrin spray was initially as effective as Olyset netting. However, whereas the netting remained nearly 100% lethal 24 h post-exposure for 12 months, the effectiveness of the sprayed insecticide declined to approximately 74% over 6–12 months. The reduction in the effectiveness of the residual spray treatment in the laboratory was similar to that observed against the cutaneous leishmaniasis vector *Lu. verrucarum* when sprayed on outside walls in Peru (Davies et al., 2000) and Olyset Plus netting has been shown to remain fully active for at least one year in field conditions (Gunay et al., 2014). It was also noted that initially the immediate mortality of the netting was significantly lower than that of the λ-cyhalothrin spray.

In field-scale evaluations of α-cypermethrin netting when used indoors was found to be durable and effective against the malaria vectors, *Anopheles culicifacies* in India (Bhatt et al., 2012) and *Anopheles* *gambiae* in Tanzania where 80% of the nets met WHOPES Phase III activity criteria at 36 months (Tungu et al., 2016). In our experiments, the α-cypermethrin netting and λ-cyhalothrin spray treatments remained in the experimental chicken sheds throughout the day and between experimental replicates were stored outside where they were exposed to UV light, fluctuating heat, humidity and rainwater. Although there is some evidence to suggest that α-cypermethrin degrades under UV light (WHO, 1989) other studies have shown that repeated exposure to UV light did not reduce the efficacy of Interceptor netting (Ouattara et al., 2013). The surface on which the insecticide is applied plays a significant role in determining the effectiveness of the insecticide treatment (Feliciangeli et al., 2003; Mutagahywa et al., 2015; Correa et al., 2019). Although the present study did not evaluate this aspect, as the netting was placed on a plywood substrate, its efficacy could potentially have been affected by either exposure to fungal growth encouraged by the proximity to damp wood or the interaction between the α-cypermethrin and the constituents of the plywood.

Experiment 3 demonstrated that when carried out in real chicken sheds, the initial effect of the netting insecticide treatment was the same as that for sprayed insecticide. Both treatments increased female sand fly mortality and significantly increased the proportion of females that were dead at 24 h but had no effect on the proportion of males dead at 24 h. This suggests that the approach of treating real chicken sheds with insecticide-treated netting to reduce numbers of female sand flies is potentially valuable. It is unclear why the treatment increased the mortality of female *Lu. longipalpis* but not males. Further long-term application studies similar to Experiment 2 are required in real chicken sheds to further evaluate their potential in killing both female and male sand flies.

In all our experiments we used 1 m^2^ of treated surface (either spray or netting). It will be important in the future to evaluate the effect of treating larger areas with the insecticides. Although spraying the interior of a real chicken shed with insecticide is feasible, lining the entire interior with netting would not be a practical option. However, doubling or quadrupling the area of insecticide-impregnated netting might bring additional relative benefits without the need to treat the whole surface area. Overall, the approach is potentially cost-effective, if the longevity of the treatment is assured, as it is simple to apply and can be combined with synthetic sex/aggregation pheromone to provide a readily accessible intervention measure for leishmaniasis control (Courtenay et al., 2019; Retkute et al., 2021).

Other studies have demonstrated the efficacy of impregnated netting and other materials against endophilic sand flies (Maroli and Majori, 1991; Alexander et al., 1995). Insecticide-impregnated curtains tested against *Phlebotomus papatasi* in Sudan almost eliminated the man-biting activity indoors. Their results clearly indicated that sand flies entering rooms provided with permethrin-impregnated curtains picked up a lethal dose of the insecticide but there was a delayed mortality effect peaking 4–8 h post-exposure (Elnaiem et al., 1999).

Our strategy, directed against exophilic *Lu. longipalpis* abundant in peridomestic environments, is a new approach. The possible population effects of the use of netting against sand flies are still unknown and more detailed studies involving bioassays and susceptibility tests comparing the same insecticides and concentrations in residual spray and impregnated nets, as well as longer lasting field experiments in sand flies aggregation sites (e.g. chicken sheds) are essential to understand the potential effectiveness of these strategies in controlling leishmaniasis. In any case, for leishmaniasis control, the regular spraying of all potential aggregation sites in Brazil, particularly in rural communities is impractical (Picado et al., 2012).

## 5 Conclusions

The main objective of these experiments was to investigate the feasibility of using netting rather than spraying as an insecticide treatment in chicken sheds alongside *Lu. longipalpis* synthetic sex aggregation pheromone. The application of insecticide-treated netting for *Lu. longipalpis* control has several potential important advantages over residual insecticide spraying; these include accurate dose control, ease of application (reduced training with no requirement for specialist spraying equipment), personnel and environmental safety, reduced costs and longevity of treatment. The application of a single piece of insecticide-impregnated netting (or other insecticide pre-treated surface) along with synthetic sex pheromone could provide a cost-effective means of *Lu. longipalpis* control. Our results indicate that netting has the potential to replace spraying as a means of delivering insecticide for vector control. However, more work is needed to understand the deterioration of the netting over time and to thus improve the long-term effectiveness of this strategy as part of a control programme applied in animal sheds and other *Lu. longipalpis* aggregation sites, in combination with synthetic sex aggregation pheromone.

## Funding

VAB was funded by the 10.13039/501100002322Coordenação de Aperfeiçoamento de Pessoal de Nível Superior (CAPES), Brazil. RPB was funded by the 10.13039/501100003593Conselho Nacional de Desenvolvimento Científico e Tecnológico (CNPq), Brazil. JGCH was funded by 10.13039/100010269The Wellcome Trust (080961/Z/06/Z), United Kingdom. The funding bodies played no role in the design of the study, or collection, analysis, and interpretation of data. They played no role in writing the manuscript.

## Ethical approval and consent to participate

The project, including the involvement of householders, was reviewed and approved by the Faculty of Health and Medicine Ethical Review Committee (FHMREC15125) at Lancaster University, UK. This study was carried out in accordance with the guidelines of the Animals in Science Regulation Unit (ASRU) and in compliance with the Animals (Scientific Procedures) Act (ASPA) 1986 (amended 2012) regulations and was consistent with UK Animal Welfare Act 2006 and The Welfare of Farmed Animals (England) Regulations 2007 and 2010. Oral consent was obtained from the Governador Valadares health authority (CCZ) to conduct the study within their administrative jurisdiction and from the householders for use of their animals and property.

## CRediT author statement

Vanessa Barbosa: Methodology, Investigation, Original Draft, Writing - Review & Editing, Visualization. Cristian de Souza: Methodology, Investigation, Writing - Review & Editing. Alisson Pereira: Investigation. Derek Gatherer: Formal analysis. Reginaldo Brazil: Conceptualization, Methodology, Resources, Writing - Original Draft, Project administration, Funding acquisition. Daniel Bray: Formal analysis, Writing - Review & Editing. James Hamilton: Conceptualization, Methodology, Resources, Writing-Original Draft, Writing-Review & Editing, Visualization, Project administration, Funding acquisition. All authors read and approved the final manuscript.

## Data availability

All data generated or analysed during this study are included in this published article and its supplementary information files.

## Declaration of competing interests

The authors declare that they have no known competing financial interests or personal relationships that could have appeared to influence the work reported in this paper.

## Appendix A Supplementary data

Multimedia component 1 **Supplementary Table S1.** The dates of the trapping experiments and location of sites used in the study. Multimedia component 1

Multimedia component 2 **Supplementary Table S2.** Control trap catches. Numbers of male and female *Lu. longipalpis* collected in HP suction traps (with light bulbs) at 2 houses without insecticide treatment. Multimedia component 2

Multimedia component 3 **Supplementary Table S3.** Numbers of male and female *Lu. longipalpis* collected in experimental chicken sheds treated with either Î±-cypermethrin impregnated netting or Î»-cyhalothrin residual spray and their mortality at 24 hours in Experiment 1. Multimedia component 3

Multimedia component 4 **Supplementary Table S4.** Numbers of male and female *Lu. longipalpis* caught in HP suction traps (fitted with pheromone but no-light) placed in experimental chicken sheds treated with either Î±-cypermethrin impregnated netting or Î»-cyhalothrin residual spray during each night and each month and their mortality after 24 hours in Experiment 2. Multimedia component 4

Multimedia component 5 **Supplementary Table S5.** Longitudinal trapping experiment: numbers of male and female *Lu. longipalpis* trapped in a pheromone baited modified HP trap in real chicken sheds treated with either Î±-cypermethrin netting or Î»-cyhalothrin residual spray in Experiment 3. Multimedia component 5
