# Supplementary material for: Insecticide-impregnated netting: A surface treatment for killing Lutzomyia longipalpis (Diptera: Psychodidae), the vector of Leishmania infantum
Source: Curr Res Parasitol Vector Borne Dis. 2021 Jul 24;1:100044. doi: 10.1016/j.crpvbd.2021.100044 (PMC8716342; doi:10.1016/j.crpvbd.2021.100044)
Supplement: Multimedia component 1 — Supplementary Table S1. The dates of the trapping experiments and location of sites used in the study. [file mmc1.docx]

**Supplementary Table S1.** The dates of the trapping experiments and location of sites used in the study.

| **Experiment** | **date** | **house** | **location** |
| --- | --- | --- | --- |
| 1 and 2 | Jan - Sept 2015 | A  B | 18°53'04.6"S 41°56'31.9"W  18°53'05.4"S 41°56'32.1"W |
| 3 | Aug - Sept 2019 | 1  2  3 | 18°53'37.53"S 41°56'35.62"W  18°53'31.53"S 41°56'25.60"W  18°52'21.24"S 41°56'2.30"W  18°52'17.44"S 41°55'55.67"W |
