# Supplementary material for: Insecticide-impregnated netting: A surface treatment for killing Lutzomyia longipalpis (Diptera: Psychodidae), the vector of Leishmania infantum
Source: Curr Res Parasitol Vector Borne Dis. 2021 Jul 24;1:100044. doi: 10.1016/j.crpvbd.2021.100044 (PMC8716342; doi:10.1016/j.crpvbd.2021.100044)
Supplement: Multimedia component 2 — Supplementary Table S2. Control trap catches. Numbers of male and female Lu. longipalpis collected in HP suction traps (with light bulbs) at 2 houses without insecticide treatment. [file mmc2.docx]

**Supplementary Table S2.** Control trap catches. Numbers of male and female *Lu. longipalpis* collected in HP suction traps (with light bulbs) at 2 houses without insecticide treatment.

|  | House A | | | | House B | | | |
| --- | --- | --- | --- | --- | --- | --- | --- | --- |
| collection  date | collected | | dead at 24h | | collected | | dead at 24h | |
|  | ♂ | **♀︎** | ♂︎ | **♀︎** | ♂︎ | **♀︎** | ♂︎ | **♀︎** |
| 26/01 | 13 | 9 | 0 | 0 | 12 | 4 | 2 | 0 |
| 27/01 | 8 | 6 | 3 | 2 | 6 | 7 | 2 | 2 |
| 28/01 | 4 | 1 | 0 | 0 | 4 | 7 | 3 | 7 |
| 29/01 | 4 | 1 | 3 | 0 | 4 | 0 | 0 | 0 |
| Total | 29 | 17 | 6 | 2 | 26 | 18 | 7 | 9 |
| $\overline{x}$ | 7.3 | 4.3 | 1.5 | 0.5 | 6.5 | 4.5 | 1.8 | 2.3 |
| ±sem | 2.1 | 2.0 | 0.9 | 0.5 | 1.9 | 1.7 | 0.6 | 1.7 |
| 12/05 | 33 | 16 | 13 | 9 | 6 | 0 | 0 | 0 |
| 13/05 | 26 | 14 | 6 | 11 | 2 | 1 | 1 | 1 |
| 14/05 | 14 | 14 | 4 | 0 | 15 | 4 | 8 | 2 |
| 15/05 | 17 | 4 | 4 | 0 | 2 | 4 | 0 | 0 |
| Total | 90 | 48 | 27 | 20 | 25 | 9 | 9 | 3 |
| $\overline{x}$ | 22.5 | 12 | 6.8 | 5 | 6.3 | 2.3 | 2.3 | 0.8 |
| ±sem | 4.3 | 2.7 | 2.1 | 2.9 | 3.1 | 1.0 | 1.9 | 0.5 |
| 07/07 | 34 | 12 | 8 | 3 | 5 | 0 | 1 | 0 |
| 08/07 | 1 | 12 | 1 | 3 | 2 | 2 | 0 | 0 |
| 09/07 | 13 | 6 | 3 | 2 | 2 | 4 | 0 | 1 |
| 10/07 | 5 | 1 | 4 | 1 | 1 | 1 | 1 | 1 |
| Total | 53 | 31 | 16 | 9 | 10 | 7 | 2 | 2 |
| $\overline{x}$ | 13 | 7.8 | 4.0 | 2.3 | 2.5 | 1.8 | 0.5 | 0.5 |
| ±sem | 7.3 | 2.7 | 1.5 | 0.5 | 0.9 | 0.9 | 0.3 | 0.3 |
| 13/09 | 4 | 2 | 2 | 1 | 3 | 1 | 1 | 0 |
| 14/09 | 2 | 2 | 0 | 0 | 8 | 3 | 0 | 0 |
| 15/09 | 2 | 0 | 1 | 0 | 4 | 4 | 1 | 1 |
| 16/09 | 1 | 1 | 0 | 0 | 9 | 5 | 2 | 0 |
| Total | 9 | 5 | 3 | 1 | 24 | 13 | 4 | 1 |
| $\overline{x}$ | 2.3 | 1.3 | 0.8 | 0.3 | 6.0 | 3.3 | 1.0 | 0.3 |
| ±sem | 0.6 | 0.5 | 0.5 | 0.3 | 1.5 | 0.9 | 0.4 | 0.3 |

Collection date was the date on which the sand flies were collected; House (A or B) is the house in which the collection was made; ♂︎ and **♀︎** collected is the number of male and female *L. longipalpis*, collected by the HP trap each night in each trap; ♂︎ and **♀︎** dead at 24h is the number of males and females that were dead after 24 hrs; total is the total number of *Lu. longipalpis* ♂︎ and **♀︎** collected during the trapping period;$\overline{x}$ is the mean number of *Lu. longipalpis* collected on each night; ±sem is ± standard error of the mean.
