# Supplementary material for: Insecticide-impregnated netting: A surface treatment for killing Lutzomyia longipalpis (Diptera: Psychodidae), the vector of Leishmania infantum
Source: Curr Res Parasitol Vector Borne Dis. 2021 Jul 24;1:100044. doi: 10.1016/j.crpvbd.2021.100044 (PMC8716342; doi:10.1016/j.crpvbd.2021.100044)
Supplement: Multimedia component 3 — Supplementary Table S3. Numbers of male and female Lu. longipalpis collected in experimental chicken sheds treated with either Î±-cypermethrin impregnated netting or Î»-cyhalothrin residual spray and their mortality at 24 hours in Experiment 1. [file mmc3.docx]

**Supplementary Table S3.** Numbers of male and female *Lu. longipalpis* collected in experimental chicken sheds treated with either α-cypermethrin impregnated netting or λ-cyhalothrin residual spray and their mortality at 24 hours in Experiment 1.

|  |  | α-cypermethrin  netting | | | | λ-cyhalothrin  spray | | | |
| --- | --- | --- | --- | --- | --- | --- | --- | --- | --- |
| N | H | collected | | dead at 24h | | collected | | dead at 24h | |
|  |  | ♂︎ | **♀︎** | ♂︎ | ♀︎ | ♂︎ | **♀︎** | ♂ | **♀︎** |
| N1 | A2 | 31 | 11 | 21 | 11 | 8 | 1 | 8 | 1 |
| N1 | B1 | 17 | 8 | 16 | 8 | 16 | 3 | 16 | 3 |
| N1 | B2 | 30 | 0 | 17 | 0 | 12 | 2 | 12 | 2 |
| N2 | A2 | 16 | 1 | 13 | 1 | 11 | 1 | 11 | 1 |
| N2 | B1 | 19 | 7 | 19 | 7 | 9 | 8 | 9 | 8 |
| N2 | B2 | 25 | 4 | 20 | 3 | 10 | 1 | 10 | 1 |
| N3 | A1 | 5 | 1 | 5 | 1 | 12 | 11 | 12 | 11 |
| N3 | A2 | 28 | 25 | 28 | 25 | 3 | 1 | 3 | 1 |
| N3 | B1 | 21 | 6 | 21 | 6 | 14 | 6 | 14 | 6 |
| N3 | B2 | 28 | 8 | 28 | 6 | 15 | 11 | 15 | 11 |
| N4 | A1 | 19 | 6 | 19 | 6 | 1 | 0 | 1 | 0 |
| N4 | A2 | 0 | 0 | 0 | 0 | 9 | 9 | 9 | 9 |
| N4 | B1 | 10 | 6 | 10 | 6 | 12 | 1 | 12 | 1 |
| N4 | B2 | 8 | 4 | 8 | 4 | 4 | 3 | 4 | 3 |
| total |  | 257 | 87 | 225 | 84 | 136 | 58 | 136 | 58 |
| $\overline{x}$ |  | 18.4 | 6.2 | 16.1 | 6.0 | 9.7 | 4.1 | 9.7 | 4.1 |
| ±sem |  | 2.6 | 1.7 | 2.2 | 1.7 | 1.2 | 1.1 | 1.2 | 1.1 |

N is the nights (1, 2, 3 or 4) on which data were collected; H is the house (A or B) and pair (1 or 2) in which the collection was made; collected ♂︎ and **♀︎** is the number of male and female *Lu. longipalpis*, collected by the HP trap (fitted with a pheromone lure and without a light) during each night in each trap; dead at 24h ♂︎ and **♀︎** is the number of males and females that were dead after 24 hrs; total is the total number of *Lu. longipalpis* ♂︎ and **♀︎** collected during the trapping period; $\overline{x}$ is the mean number collected on each night; ±sem is ± standard error of the mean.
