# Supplementary material for: Insecticide-impregnated netting: A surface treatment for killing Lutzomyia longipalpis (Diptera: Psychodidae), the vector of Leishmania infantum
Source: Curr Res Parasitol Vector Borne Dis. 2021 Jul 24;1:100044. doi: 10.1016/j.crpvbd.2021.100044 (PMC8716342; doi:10.1016/j.crpvbd.2021.100044)
Supplement: Multimedia component 4 — Supplementary Table S4. Numbers of male and female Lu. longipalpis caught in HP suction traps (fitted with pheromone but no-light) placed in experimental chicken sheds treated with either Î±-cypermethrin impregnated netting or Î»-cyhalothrin residual spray during each night and each month and their mortality after 24 hours in Experiment 2. [file mmc4.docx]

**Supplementary Table S4.** Numbers of male and female *Lu. longipalpis* caught in HP suction traps (fitted with pheromone but no-light) placed in experimental chicken sheds treated with either α-cypermethrin impregnated netting or λ-cyhalothrin residual spray during each night and each month and their mortality after 24 hours in Experiment 2.

| MAY |  | α-cypermethrin  netting | | | | λ-cyhalothrin  spray | | | |
| --- | --- | --- | --- | --- | --- | --- | --- | --- | --- |
| N | H | collected | | dead at 24h | | collected | | dead at 24h | |
|  |  | ♂︎ | ♀︎ | ♂︎ | ♀︎ | ♂︎ | ♀︎ | ♂︎ | ♀︎ |
| N1 | A1 | 17 | 12 | 17 | 12 | 20 | 10 | 20 | 10 |
| N1 | A2 | 115 | 30 | 116 | 27 | 14 | 11 | 14 | 11 |
| N2 | A1 | 17 | 13 | 17 | 13 | 13 | 8 | 13 | 8 |
| N2 | A2 | 39 | 13 | 37 | 9 | 14 | 11 | 14 | 11 |
| N2 | B1 | 10 | 9 | 9 | 9 | 6 | 7 | 6 | 7 |
| N2 | B2 | 6 | 4 | 6 | 4 | 16 | 7 | 16 | 7 |
| N3 | B1 | 10 | 5 | 10 | 5 | 8 | 0 | 8 | 0 |
| N3 | B2 | 15 | 8 | 15 | 8 | 11 | 0 | 11 | 0 |
| N4 | A1 | 16 | 4 | 15 | 4 | 7 | 1 | 7 | 1 |
| N4 | A2 | 16 | 3 | 16 | 3 | 9 | 4 | 9 | 4 |
| N4 | B1 | 9 | 2 | 9 | 2 | 16 | 5 | 16 | 5 |
| N4 | B2 | 9 | 4 | 9 | 4 | 17 | 3 | 17 | 3 |
| Total |  | 280 | 107 | 276 | 100 | 151 | 67 | 151 | 67 |
| $\overline{x}$ |  | 23.3 | 8.9 | 23.0 | 8.3 | 12.6 | 5.6 | 12.6 | 5.6 |
| ±sem |  | 8.8 | 2.2 | 8.8 | 2.0 | 1.3 | 1.2 | 1.3 | 1.2 |

| JULY |  | α-cypermethrin  netting | | | | λ-cyhalothrin  spray | | | |
| --- | --- | --- | --- | --- | --- | --- | --- | --- | --- |
| N | H | collected | | dead at 24h | | collected | | dead at 24h | |
|  |  | ♂︎ | ♀︎ | ♂︎ | ♀︎ | ♂︎ | ♀︎ | ♂︎ | ♀︎ |
| N1 | A1 | 34 | 17 | 32 | 16 | 10 | 5 | 10 | 5 |
| N1 | A2 | 44 | 9 | 43 | 7 | 40 | 14 | 40 | 14 |
| N1 | B1 | 10 | 4 | 10 | 3 | 19 | 3 | 19 | 3 |
| N1 | B2 | 20 | 2 | 17 | 2 | 29 | 6 | 29 | 6 |
| N2 | A1 | 15 | 3 | 15 | 3 | 14 | 11 | 14 | 11 |
| N2 | A2 | 38 | 13 | 32 | 11 | 13 | 11 | 13 | 10 |
| N3 | A1 | 16 | 8 | 16 | 8 | 7 | 5 | 7 | 5 |
| N3 | A2 | 41 | 10 | 41 | 10 | 39 | 19 | 38 | 19 |
| N3 | B1 | 10 | 2 | 10 | 2 | 14 | 5 | 14 | 5 |
| N3 | B2 | 1 | 2 | 1 | 2 | 13 | 7 | 13 | 7 |
| N4 | A1 | 14 | 11 | 14 | 10 | 13 | 9 | 13 | 9 |
| N4 | A2 | 63 | 20 | 63 | 20 | 32 | 5 | 32 | 5 |
| N4 | B1 | 22 | 4 | 18 | 4 | 0 | 0 | 0 | 0 |
| N4 | B2 | 11 | 3 | 11 | 3 | 13 | 7 | 13 | 7 |
| Total |  | 339 | 108 | 323 | 101 | 256 | 107 | 255 | 106 |
| $\overline{x}$ |  | 24.2 | 7.7 | 23.1 | 7.2 | 18.3 | 7.6 | 18.2 | 7.6 |
| ±sem |  | 4.6 | 1.6 | 4.5 | 1.5 | 3.2 | 1.3 | 3.2 | 1.3 |

| SEPT |  | λ-cypermethrin  netting | | | | λ-cyhalothrin  spray | | | |
| --- | --- | --- | --- | --- | --- | --- | --- | --- | --- |
| N | H | collected | | dead at 24h | | collected | | dead at 24h | |
|  |  | ♂︎ | ♀︎ | ♂︎ | ♀︎ | ♂︎ | ♀︎ | ♂︎ | ♀︎ |
| N1 | A1 | 16 | 7 | 16 | 7 | 3 | 6 | 3 | 6 |
| N1 | A2 | 30 | 14 | 20 | 12 | 31 | 19 | 30 | 18 |
| N1 | B1 | 38 | 6 | 18 | 0 | 18 | 5 | 18 | 5 |
| N1 | B2 | 2 | 1 | 2 | 1 | 46 | 14 | 26 | 10 |
| N2 | A1 | 3 | 0 | 2 | 0 | 8 | 7 | 8 | 6 |
| N2 | A2 | 18 | 9 | 7 | 2 | 3 | 7 | 3 | 7 |
| N2 | B1 | 12 | 4 | 9 | 1 | 14 | 2 | 14 | 2 |
| N2 | B2 | 17 | 17 | 14 | 3 | 2 | 0 | 2 | 0 |
| N3 | A1 | 14 | 19 | 11 | 12 | 2 | 1 | 2 | 1 |
| N3 | A2 | 19 | 12 | 17 | 9 | 12 | 10 | 12 | 10 |
| N3 | B1 | 18 | 1 | 8 | 1 | 7 | 3 | 7 | 2 |
| N3 | B2 | 2 | 0 | 2 | 0 | 16 | 6 | 16 | 5 |
| N4 | A1 | 0 | 0 | 0 | 0 | 5 | 5 | 5 | 5 |
| N4 | A2 | 18 | 11 | 14 | 2 | 12 | 15 | 12 | 14 |
| N4 | B1 | 7 | 0 | 6 | 0 | 5 | 1 | 5 | 1 |
| N4 | B2 | 17 | 2 | 16 | 1 | 0 | 0 | 0 | 0 |
| total |  | 231 | 103 | 162 | 51 | 184 | 101 | 163 | 92 |
| $\overline{x}$ |  | 14.4 | 6.4 | 10.1 | 3.2 | 11.5 | 6.3 | 10.2 | 5.6 |
| ±sem |  | 2.6 | 1.6 | 1.6 | 1.1 | 3.0 | 1.4 | 2.2 | 1.3 |

N is the nights (1, 2, 3 or 4) on which data were collected; H is the house (A or B) and pair (1 or 2) in which the collection was made; ♂︎ and **♀︎** collected is the number of *Lu. longipalpis*, male and female collected by the HP trap during each night in each trap; ♂ and **♀︎** dead at 24h is the number of males and females that were dead after 24 hrs; total is the total number of *Lu. longipalpis* ♂︎ and **♀︎** collected during the trapping period; $\overline{x}$ is the mean number of *Lu. longipalpis* collected on each night; ± sem is ± standard error of the mean.
