# Supplementary material for: Insecticide-impregnated netting: A surface treatment for killing Lutzomyia longipalpis (Diptera: Psychodidae), the vector of Leishmania infantum
Source: Curr Res Parasitol Vector Borne Dis. 2021 Jul 24;1:100044. doi: 10.1016/j.crpvbd.2021.100044 (PMC8716342; doi:10.1016/j.crpvbd.2021.100044)
Supplement: Multimedia component 5 — Supplementary Table S5. Longitudinal trapping experiment: numbers of male and female Lu. longipalpis trapped in a pheromone baited modified HP trap in real chicken sheds treated with either Î±-cypermethrin netting or Î»-cyhalothrin residual spray in Experiment 3. [file mmc5.docx]

**Supplementary Table S5.** Longitudinal trapping experiment: numbers of male and female *Lu.* *longipalpis* trapped in a pheromone baited modified HP trap in real chicken sheds treated with either α-cypermethrin netting or λ-cyhalothrin residual spray in Experiment 3.

|  | before intervention | | | | after intervention | | | | | | | |
| --- | --- | --- | --- | --- | --- | --- | --- | --- | --- | --- | --- | --- |
|  | no insecticide | | | | α-cypermethrin netting | | | | λ-cyhalothrin spray | | | |
| H | collected | | dead at 24h | | collected | | dead at 24h | | collected | | dead at 24h | |
|  | ♂︎ | ♀︎ | ♂︎ | ♀︎ | ♂︎ | ♀︎ | ♂︎ | ♀︎ | ♂︎ | ♀ | ♂︎ | ♀︎ |
| 1 | 35 | 13 | 22 | 5 | - | - | - | - | 35 | 13 | 18 | 8 |
| 2 | 3 | 0 | 2 | 0 | - | - | - | - | 6 | 3 | 4 | 1 |
| 3 | 13 | 5 | 8 | 1 | - | - | - | - | 17 | 7 | 10 | 5 |
| 4 | 15 | 17 | 3 | 0 | - | - | - | - | 29 | 11 | 17 | 4 |
| 2 | 8 | 0 | 2 | 0 | - | - | - | - | 1 | 1 | 0 | 1 |
| 4 | 23 | 19 | 17 | 10 | - | - | - | - | 62 | 18 | 40 | 11 |
| 1 | 26 | 11 | 20 | 8 | - | - | - | - | 39 | 12 | 38 | 12 |
| 3 | 7 | 1 | 4 | 1 | - | - | - | - | 22 | 5 | 19 | 5 |
| 1 | 19 | 6 | 17 | 5 | - | - | - | - | 14 | 13 | 14 | 13 |
| 3 | 7 | 4 | 2 | 3 | - | - | - | - | 12 | 2 | 7 | 1 |
| 3 | 10 | 2 | 2 | 2 | 10 | 3 | 8 | 3 | - | - | - | - |
| 4 | 32 | 8 | 14 | 4 | 8 | 6 | 5 | 4 | - | - | - | - |
| 1 | 86 | 9 | 39 | 3 | 28 | 7 | 27 | 3 | - | - | - | - |
| 2 | 20 | 7 | 19 | 7 | 1 | 1 | 0 | 1 | - | - | - | - |
| 1 | 67 | 15 | 40 | 9 | 18 | 5 | 18 | 5 | - | - | - | - |
| 3 | 19 | 10 | 13 | 6 | 10 | 3 | 8 | 3 | - | - | - | - |
| 2 | 6 | 0 | 5 | 0 | 3 | 1 | 1 | 1 | - | - | - | - |
| 4 | 26 | 17 | 18 | (0 | 23 | 12 | 23 | 12 | - | - | - | - |
| 2 | 2 | 2 | 2 | 2 | 1 | 2 | 0 | 1 | - | - | - | - |
| 4 | 9 | 3 | 2 | 1 | 16 | 11 | 12 | 8 | - | - | - | - |
| total^+^ | 433 | 149 | 251 | 77 |  |  |  |  |  |  |  |  |
| mean | 21.7 | 7.5 | 12.6 | 3.9 |  |  |  |  |  |  |  |  |
| ±sem | 21.2 | 6.2 | 11.7 | 3.5 |  |  |  |  |  |  |  |  |
| total* | 277 | 73 | 154 | 44 | 118 | 51 | 102 | 41 |  |  |  |  |
| mean | 27.7 | 7.3 | 15.4 | 4.4 | 11.8 | 5.1 | 10.2 | 4.1 |  |  |  |  |
| ±sem | 8.8 | 1.8 | 4.5 | 1.1 | 2.9 | 1.2 | 3.1 | 1.1 |  |  |  |  |
| total^#^ | 156 | 76 | 97 | 33 |  |  |  |  | 237 | 85 | 167 | 61 |
| mean | 15.6 | 7.6 | 9.7 | 3.3 |  |  |  |  | 23.7 | 8.5 | 16.7 | 6.1 |
| ±sem | 3.2 | 2.2 | 2.6 | 1.1 |  |  |  |  | 5.7 | 1.8 | 4.2 | 1.5 |

R is the experimental replicate; H is the house in which the chicken shed was located; before intervention is the number of male and female *Lu. longipalpis* collected when no insecticide treatment was present (1^st^ night); after intervention is the number of male and female *Lu. longipalpis* collected when either α-cypermethrin netting or λ-cyhalothrin spray insecticide treatment was present in the chicken shed (2^nd^ night). Total^+^ is the total number of sand flies collected and dead after 24h in all chicken sheds and on all nights prior to intervention. Total* is the total number of sand flies collected and dead after 24h in all chicken sheds and on all nights after the α-cypermethrin netting intervention and total^#^ is the total number of sand flies collected and dead after 24h in all chicken sheds and on all nights after the λ-cyhalothrin spray intervention.
